# Generalization optimizing machine learning to improve CT scan radiomics and assess immune checkpoint inhibitors’ response in non-small cell lung cancer: a multicenter cohort study

**DOI:** 10.3389/fonc.2023.1196414

**Published:** 2023-07-20

**Authors:** Marion Tonneau, Kim Phan, Venkata S. K. Manem, Cecile Low-Kam, Francis Dutil, Suzanne Kazandjian, Davy Vanderweyen, Justin Panasci, Julie Malo, François Coulombe, Andréanne Gagné, Arielle Elkrief, Wiam Belkaïd, Lisa Di Jorio, Michele Orain, Nicole Bouchard, Thierry Muanza, Frank J. Rybicki, Kam Kafi, David Huntsman, Philippe Joubert, Florent Chandelier, Bertrand Routy

**Affiliations:** ^1^ Department of Cancer Research, Centre de Recherche du Centre Hospitalier Universitaire de Montréal (CRCHUM), Montreal, QC, Canada; ^2^ Université de Médecine, Lille, France; ^3^ Imagia Canexia Health, Montreal, QC, Canada; ^4^ Institut Universitaire de Cardiologie et de Pneumologie de Quebec, Université Laval, Québec City, QC, Canada; ^5^ Department of Mathematics and Computer Science, University of Quebec at Trois-Rivières, Trois-Rivières, QC, Canada; ^6^ Department of Medical Oncology, Jewish General Hospital, Montreal, QC, Canada; ^7^ Department of Radiology, Centre Hospitalier de Sherbrooke (CHUS), Sherbrooke, QC, Canada; ^8^ Hemato-Oncology Division, Centre Hospitalier de l’université de Montreal, Montreal, QC, Canada; ^9^ Department of Oncology, Centre Hospitalier de Sherbrooke (CHUS), Sherbrooke, QC, Canada; ^10^ Department of Radiation Oncology, Lady Davis Institute of the Jewish General Hospital, Montreal, QC, Canada; ^11^ Department of Pathology, Institut Universitaire de Cardiologie et de Pneumologie de Québec, Québec, QC, Canada

**Keywords:** radiomics, Deeplearning, NSCLC, immunotherapy, DeepRadiomics

## Abstract

**Background:**

Recent developments in artificial intelligence suggest that radiomics may represent a promising non-invasive biomarker to predict response to immune checkpoint inhibitors (ICIs). Nevertheless, validation of radiomics algorithms in independent cohorts remains a challenge due to variations in image acquisition and reconstruction. Using radiomics, we investigated the importance of scan normalization as part of a broader machine learning framework to enable model external generalizability to predict ICI response in non-small cell lung cancer (NSCLC) patients across different centers.

**Methods:**

Radiomics features were extracted and compared from 642 advanced NSCLC patients on pre-ICI scans using established open-source PyRadiomics and a proprietary DeepRadiomics deep learning technology. The population was separated into two groups: a discovery cohort of 512 NSCLC patients from three academic centers and a validation cohort that included 130 NSCLC patients from a fourth center. We harmonized images to account for variations in reconstruction kernel, slice thicknesses, and device manufacturers. Multivariable models, evaluated using cross-validation, were used to estimate the predictive value of clinical variables, PD-L1 expression, and PyRadiomics or DeepRadiomics for progression-free survival at 6 months (PFS-6).

**Results:**

The best prognostic factor for PFS-6, excluding radiomics features, was obtained with the combination of Clinical + PD-L1 expression (AUC = 0.66 in the discovery and 0.62 in the validation cohort). Without image harmonization, combining Clinical + PyRadiomics or DeepRadiomics delivered an AUC = 0.69 and 0.69, respectively, in the discovery cohort, but dropped to 0.57 and 0.52, in the validation cohort. This lack of generalizability was consistent with observations in principal component analysis clustered by CT scan parameters. Subsequently, image harmonization eliminated these clusters. The combination of Clinical + DeepRadiomics reached an AUC = 0.67 and 0.63 in the discovery and validation cohort, respectively. Conversely, the combination of Clinical + PyRadiomics failed generalizability validations, with AUC = 0.66 and 0.59.

**Conclusion:**

We demonstrated that a risk prediction model combining Clinical + DeepRadiomics was generalizable following CT scan harmonization and machine learning generalization methods. These results had similar performances to routine oncology practice using Clinical + PD-L1. This study supports the strong potential of radiomics as a future non-invasive strategy to predict ICI response in advanced NSCLC.

## Introduction

The recent advent of radiomics by quantitative image analysis has been gaining interest in oncology as a novel strategy for cancer screening and predicting treatment response (1). Immune checkpoint inhibitors (ICIs) represent the standard of care for patients with advanced non-small cell lung cancer (NSCLC), and development of biomarkers represents a paramount interest (2–4). Nevertheless, primary resistance to ICIs remains unpredictable, reaching up to 60%, while the rate of secondary resistance approaches 100% (5–7). Assessment of PD-L1 expression in tumor tissue has been widely used to determine the therapeutic approach of either a single-agent anti-PD-1 inhibitor or the combination of platinum doublet with anti-PD-1 for patients with tumor PD-L1 expression ≥50% or <50%, respectively (4, 8).

Radiomics has been shown to predict CD8^+^ T-cell infiltration and response to ICIs or radiotherapy (9–12). Additional studies attempted to determine PD-L1 expression, the only approved predictive biomarker in advanced NSCLC (13, 14). Nevertheless, validation of radiomic models requires large image datasets that include different cancer centers and a variety of computed tomography (CT) scanners. The necessary diversity is a major hurdle to validate published radiomics signatures in independent cohorts (15). Different image acquisition parameters and different reconstruction kernels with varying slice thicknesses alter the predictive potential of radiomics (16). Therefore, the development of signatures applicable across academic centers is a challenge that has stymied the adoption of radiomics in routine oncology clinical practice. Altogether, this highlights the importance of harmonizing image acquisition and reconstruction procedures to reduce multicenter variability before gathering data (17, 18). In recent years, research efforts have focused on developing a statistical harmonization strategy called ComBat (18–20). ComBat acts directly on already computed features, not on the original images. While a privacy advantage on one hand, this method is only capable of harmonizing for a single batch effect at a time; as further detailed (21), if variance in image acquisition and reconstruction protocols affects image properties, then different batches should be used for the same scanner corresponding to different settings. Furthermore, four assumptions have to be met for ComBat to generate valid results among which (1) covariates (if any) that might explain different distributions at two or more sites have to be identified and considered to redesign the original ComBat approach (2); the different sets of feature values to be realigned have to be independent, which challenges the very use of PyRadiomics known for many correlating features; and (3) determining a single transformation with ComBat from data with different tissue or tumor types does not always lead to satisfactory data realignments, because different texture patterns are not necessarily affected identically by the image acquisition and reconstruction protocols (21). Taking these constraints altogether, the present study hypothesized that developing specific machine learning generalization methods rather than applying more complex feature harmonization strategies might prove more successful.

In this study, using conventional harmonization techniques together with AI generalization strategies, we showed that a radiomic signature generated in a discovery cohort from three independent cancer centers of NSCLC patients amenable to ICI to predict PFS at 6 months could be validated in a fourth cohort. This new method designed for generalizability rather than traditional performance has the potential to further the use of radiomics in routine oncology practice.

## Methods

### Study population

This retrospective study included 642 advanced NSCLC patients treated with anti-PD-1 alone or in combination with platinum-doublet chemotherapy between 2015 and 2021 in the chemotherapy-refractory or first-line settings. Signed, informed consent was obtained from each patient, and the study was approved by the Institutional Review Board [Human Ethics Committee (MP-02-2019-8091)] at four academic institutions where patient data were acquired: Centre Hospitalier de l’Université de Montréal (CHUM), Jewish General Hospital in Montreal (JGH), Institut Universitaire de Cardiologie et Pneumologie de Québec – Université Laval (IUCPQ-UL), and Centre Hospitalier de l’Université de Sherbrooke (CHUS). All patients with histology-proven stage III or IV NSCLC treated with ICI and with a pre-ICI CT scan were eligible for retrospective review. Response Evaluation Criteria in Solid Tumors (RECIST) criteria version 1.1 was used to assess tumor response, and all patients were followed until death or until the data were locked on 15 January 2022 (22).

### Clinical data analysis

We separated the total population into two independent cohorts. All patients from CHUM, JGH, and IUCPQ comprised the discovery cohort (*n* = 512 patients) while patients from CHUS comprised the validation cohort (*n* = 130 patients). Baseline demographic and clinicopathological characteristics were compared between the discovery and validation cohort using chi-square or Fisher’s exact test for categorical variables and Student’s *t*-test or Mann–Whitney *U* test for continuous variables, as appropriate. Clinical outcome of PFS at 6 months was used as the stronger outcome marker for NSCLC patients amenable to ICIs as this clinical marker was found to be one of the most robust (3). All patients included had a PFS superior to 6 months or progressed before.

### Harmonization process

#### CT scan normalization

**Figure 1:**
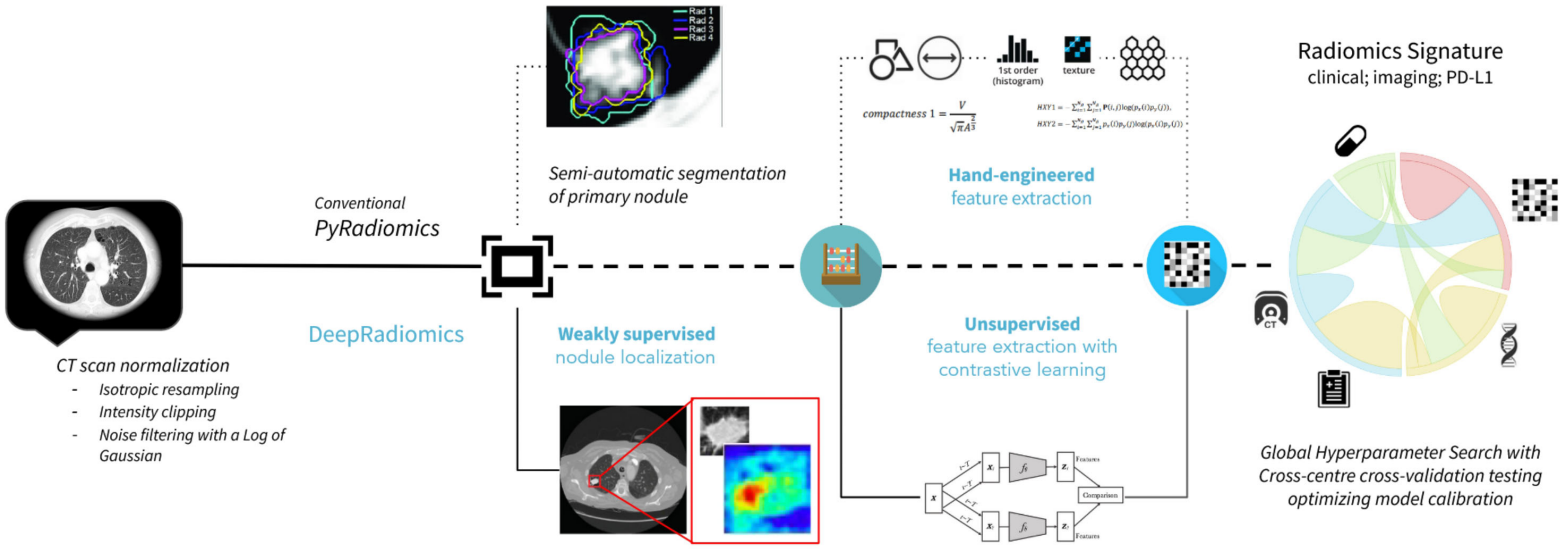
Radiomics workflows used in the study. The upper panel represents PyRadiomics pipeline including, sequentially, the segmentation input and hand-engineered feature extractions. The lower panel represents the DeepRadiomics pipeline with weakly supervised region extractions and automated feature learning extractions.

Each primary lesion was manually annotated by a radiation oncologist or a radiologist for identifying the tumor’s longest axis, on de-identified, pretreatment CT images. The following pre-processing steps were applied to all scans: resampling to 1-mm isometric voxels (to normalize pixel and slice thickness variation) followed by Hounsfield Unit (HU) truncation to a range −400 HU to 1,024 HU (to reduce the impact of artifacts on radiomics features), followed by image noise normalization using a Laplacian of Gaussian filter from the PyRadiomics library with hyperparameter values for sigma2 = {1,3} (20, 23) (see **Figure 1**).

#### Radiomics feature extraction

##### PyRadiomics features extraction

We used a three-stage process to determine the region of interest (ROI) used for extracting radiomic features. The first stage consisted of CT scan alignment achieved by principal component analysis (PCoA), followed by chest isolation through mathematical morphology-based denoising, and finally chest segmentation based on connected regions (24). In the second stage, the lung was automatically segmented based on the detected skin boundary, rough segmentation of lung contour, and pulmonary parenchyma refinement. Next, this lung segmentation was intersected with a clustering-based nodule mask to identify a nodule ROI agnostic of size, position, and spreading near or through the pleura, utilizing the relative symmetry of the lung (25). These ROIs were then assessed for clinical appropriateness in view of known shortcomings of segmentation techniques reported in the radiomics literature that extend beyond the objective of the current study but for which, nonetheless, we present an alternative in the form of a new DeepRadiomics method. From the segmentation, the PyRadiomics library v3.0.1, an open-source python package for the extraction of radiomics features from medical imaging, was used to extract 94 candidate radiomics features: 19 first-order features and 75 second-order features with a Laplacian of Gaussian (LoG) filter applied (26). The reproducibility of extracting PyRadiomics features from different segmentations has been well studied, and a high reproducibility was reported for first-order, Laplacian, Gaussian-filtered features and texture features, but low reproducibility for shape and wavelet features. Indeed, wavelet features were extracted but omitted from analyses due to the high association with the acquisition parameters.

##### DeepRadiomics features extraction

We proposed a new data-driven alternative to the traditional PyRadiomics method. We followed the emerging interest in deep learning models to provide suitable high-throughput extraction of quantitative imaging features from medical images (27, 28). A VGG16 backbone was pre-trained to learn image features followed by a SimCLR process, a self-learning framework for contrastive learning of visual representations (29, 30). The pre-training datasets consisted of the public dataset [Lung Image Database Consortium (LIDC)], after applying the CT scan normalization procedure described in this article (31), in order to leverage a larger dataset of lung CT scans also obtained across multiple institutions and acquisition parameters. The pretraining was made on 2D patches of 48 × 48 pixels, centered on the nodules. For each image in a batch, we applied two different sets of data augmentation (random translation, rotation, flip, gaussian blur, and zoom), while training the model to correctly identify pairs of images representing the same nodule among other nodules in the same batch. In our proposed method, relevant images were processed using the pretrained SimCLR network, and automatically learned features were extracted from its last convolutional layer. Patch input could not be smaller than 32 × 32 due to the model architecture backbone, so we expanded context as needed for ROIs smaller than this. Finally, the full ROI bounding box derived from the annotation process was used instead of segmenting or delineating potential lesions as is usually required in radiomics, which we see as further contributing to clinical generalization.

##### Toward the generalizability of AI models across healthcare settings

We designed a global hyperparameter search framework (the “GHPS”) to autonomously determine the final artificial intelligence (AI) model based on generalization-optimizing parameter configurations rather than performance-optimizing configurations. GHPS is ideally implemented by iteratively performing cross-center cross-validation testing over all combinations of parameters composed of the following four methods (32): (a) feature extraction, (b) feature selection, (c) model selection, and (d) model hyperparameter tuning. To reduce computational complexity, we elected to perform cross-validation testing, after processing all data with our normalization strategy, over all combinations of parameters consisting of (i) feature selection, (ii) model selection, and (iii) model hyperparameter tuning. Finally, our cross-validation testing allowed for refining the estimated final model with the best average performance remaining within small cross-center variability in performance, thus ensuring optimal generalizability.

We used a Sobol sequence for the randomized hyperparameter search to construct low discrepancy sets (33). The feature selection space was optimized for removing highly correlated features using a Spearman rho method with thresholds ranging from 0.8 to 1, as well as evaluating the optimal feature reduction method from among (a) *F*-test, (b) three relief-based algorithms from the open-source library ReBATE (34), or (c) a custom implementation of the Maximum Relevance − Minimum Redundancy strategy (35). Model selection space was optimized for identifying non-overfitting methods on our datasets (across folds) from logistic regression and XGboost, and then defining the optimal parametrization of such, considering (a) a metric of calibration with Nagekjerje’s R index (average), (b) metrics of discrimination with the area under the curve (AUC), and (c) a metric of goodness of fit with the Brier’s score; altogether, the agreement of these metrics is chosen as a proxy for generalizability due to their ability to capture (correlate with) the variance of the AUC (**Supplementary Table 1**). Moreover, we extended this observation by measuring the Youden’s *J* statistics from both the discovery and validation models where the Clinical + DeepRadiomics model had a superior Youden score compared to Clinical + PD-L1 or Clinical + PyRadiomics, informing on the probability of a model to support an informed decision as opposed to a random guess, taking into account all model predictions (**Supplementary Table 2**).

The model with both best average and smallest variability in performance across folds was selected as the final model. The hyperparameter tuning space of the “selected model” was optimized for discovering the final 5 to 20 features best representing the complete information space (at each fold), inclusive of a set of 5 fixed features comprising lesion radius, ECOG score, age, smoking history (never/former/current), and first-line ICI (yes/no), with and without including PD-L1 status. In that manner, imaging features would only be identified in case of complementing clinically relevant features as per our primary objective.

We used the bootstrap 95% confidence interval of the model performance in the discovery cohort for establishing a judgment of generalizability to the validation cohort (36). We determined the success of a biomarker’s generalizability test when its estimate of the AUC derived from the validation cohort fell within the confidence interval of estimate of the AUC derived from the discovery cohort.

## Results

### Cohort description

This study included a total of 910 eligible patients from four institutions. A first selection was made on availability of imaging within 3 months prior to initiation of ICI therapy, which resulted in the exclusion of 147 patients. Then, we excluded 121 patients for which a primary lesion could not be clearly delineated in the annotation process, to reach a final population of 642 patients (**Supplementary Figure 1**). A total of 512 patients from the discovery cohort had a median PFS of 5.5 months (95% CI [4.8–6.7]) and 130 patients from the validation cohort had a median PFS of 6.1 months (95% CI [5.1–7.5]) (*p* = 0.377). There were no statistical differences between the discovery and validation cohorts regarding the mean age, sex proportion, history of smoking, distribution of ECOG status, stage, or distribution of the PD-L1 group (all *p* > 0.05) (**Table 1**). However, a larger proportion of patients in the validation cohort received ICI as first-line therapy (72%) compared to the discovery cohort (39%) (*p* < 0.001) (**Table 1**). With respect to outcome, the proportion of patients in discovery and validation cohorts with PFS at 6 months were 51% and 46%, respectively (*p* = 0.377).

**Table 1:** Baseline characteristics of 642 patients segregated into the discovery and validation cohorts.

	Discovery cohort n=512	Validation cohort n=130	*p-value*
Age - median [IQR]	68.3 [62.3, 73.8]	67.1 [60.5, 72.8]	*0.232*
**Sex** - n (%) Male Female	258 (51) 254 (49)	69 (53) 61 (47)	* * *0.653*
**Smoking history** - n (%) Current or former Never	466 (92) 46 (8)	125 (97) 5 (3)	* * *0.067*
**ECOG status** - n (%) 0 1 >2	137 (27) 292 (59) 68 (14)	37 (29) 77 (59) 16 (12)	* * *0.514*
**Stage** - n (%) III IV	52 (10) 460 (90)	11 (8) 119 (92)	* * *0.642*
**Histology** - n (%) Adenocarcinoma Squamous Other	404 (79) 29 (6) 79 (15)	89 (69) 13 (10) 28 (21)	* * *0.034*
**PD-L1 status** - n (%) <1% 1-49% ≥50%	97 (22) 117 (27) 223 (51)	36 (29.0) 34 (27.4) 54 (43.5)	* * *0.222*
**Line of treatment** - n (%) First line Second line or more	197 (39) 315 (61)	94 (72) 36 (28)	* * *<0.001*
**Type of treatment** - n (%) ICI alone Chemotherapy-ICI	469 (91) 43 (9)	76 (59) 54 (41)	* * *<0.001*
**Progression-free survival** - n (%) ≥ 6 months < 6 months	260 (51) 252 (49)	60 (46) 70 (54)	* * *0.377*

Italic terms define the p-value.

### Benchmark of clinical outcome prediction with standard clinicopathological features

First, we sought to define the role of standard-of-care prognostic score using only clinical variables (age, ECOG status, smoking status, and line of treatment) alone or in combination with PD-L1 expression to establish a benchmark for clinical outcome prediction. The best clinical prognostic factor for PFS-6 was obtained with the combination of Clinical + PD-L1 expression with an AUC of 0.66 (95% CI [0.61–0.70]) in the discovery cohort and 0.62 (95% CI [0.53–0.72]) in the validation cohort (**Supplementary Figures 2A, D**). Clinical markers alone did not perform as well with an AUC of 0.64 (95% CI [0.59–0.69]) and 0.58 (95% CI [0.49–0.69]) in the discovery and validation cohort, respectively. Similarly, the AUC of the model with PD-L1 expression alone was 0.56 (95% CI [0.52–0.61]) in the discovery cohort and 0.59 (95% CI [0.48–0.70]) in the validation cohort (**Supplementary Figures 2B, C, E, F**).

### Radiomics prediction without harmonization

**Figure 2:**
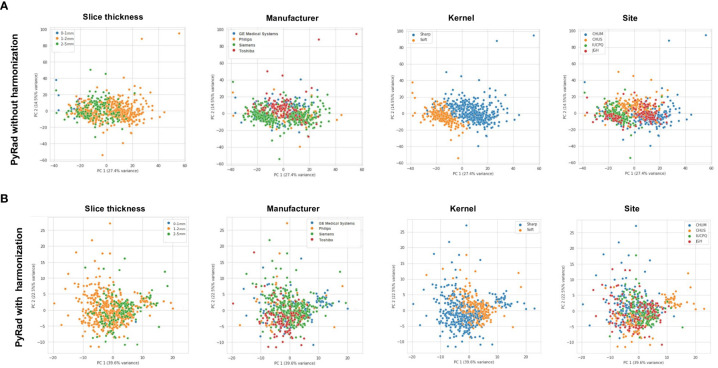
**(A)** Principal component analysis (PCoA) of PyRadiomics representation of various CT-scan acquisition parameters (Slide thickness, Manufacturer, Kernel, and Site) prior to image harmonization. **(B)** PCoA of PyRadiomics representation of various CT-scan acquisition parameters (Slide thickness, Manufacturer, Kernel, and Site) after image harmonization. PyRad, PyRadiomics.

**Figure 3:**
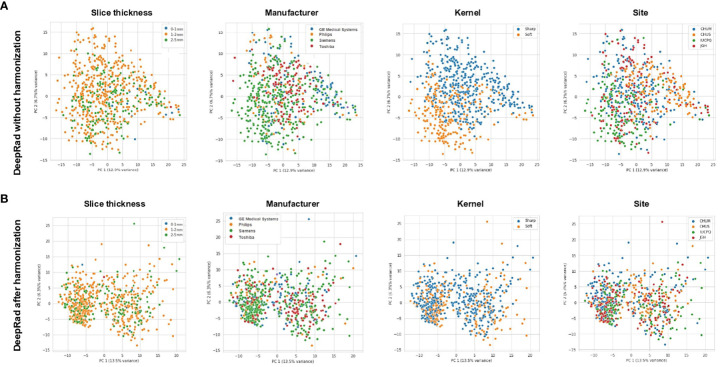
**(A)** Principal component analysis (PCoA) of DeepRadiomics representation of various CT-scan acquisition parameters (Slide thickness, Manufacturer, Kernel, and Site) prior to image harmonization. **(B)** PCoA of DeepRadiomics representation of various CT-scan acquisition parameters (Slide thickness, Manufacturer, Kernel, and Site) after image harmonization. DeepRad, DeepRadiomics.

Subsequently, we measured the predictive role of radiomics with no harmonization. We used PCoA to facilitate the projection and visualization of high-dimensional radiomic feature data. Before data harmonization, using PyRadiomic features, we observed clustering by CT scan slice thickness, manufacturer, kernel, and academic centers (**Figure 2A**). PCoA obtained from DeepRadiomics revealed similar clustering effect across the different medical centers, CT vendors, reconstruction kernels, and slice thicknesses (**Figure 3A**). These clusters were expected based on the important difference in CT acquisition parameters from each center (**Supplementary Table 3**). Without CT harmonization, a model combining Clinical + PyRadiomics or DeepRadiomics features to predict PFS-6 featured had an AUC of 0.69 (95% CI [0.64–0.74]) and 0.69 (95% CI [0.64–0.74]), respectively, in the discovery cohort (**Supplementary Figure 3A**). Nevertheless, AUC in the validation cohorts did not generalize; the AUC was 0.57 for Clinical + PyRadiomics and 0.52 for Clinical + DeepRadiomics both outside their respected interval CI obtained in the discovery cohorts (**Supplementary Figure 3B**).

Next, we aimed to include PD-L1 to these models to improve generalizability. First, PD-L1 did not increase the AUC in the discovery cohorts for both radiomic signatures with AUC reaching 0.71 compared to 0.69 without PD-L1 (**Supplementary Figure 3C**). Second, the addition of PD-L1 did not support the generalizability of performances in the validation cohorts (**Supplementary Figure 3D**).

### Image processing-based harmonization of radiomics features

Following the normalization of raw CT scans data detailed in the “CT scan normalization” section, and processing first for PyRadiomics, we obtained a relatively homogeneous population for the clusters of slice thickness, manufacturer, and site (**Figure 2B**). Despite improvement in kernel distribution on the PCoA, visual clustering was still observed.

Next, the same normalization method alongside DeepRadiomics revealed a broad homogenization across the four parameters of interest including kernel (**Figure 3B**). Of note, we observed, after normalization, two populations across all PCoA. After further investigation, we confirmed that these populations were a result of the patch size of 48 × 48 pixels used during pretraining of the VGG16 backbone introduced in the “DeepRadiomics features extraction” section (**Supplementary Figure 4A**). To assess if the radius clusters were confounders for PFS-6 months, we represented a PCoA for DeepRadiomics features vs. PFS-6 months (**Supplementary Figure 4B**). We observed that there was no association of radius cluster with PFS-6 months. Our results showed that, after normalization, we were able to mitigate for variation across medical centers, CT vendors, acquisition variability, and reconstruction kernels.

### Performance of AI-based radiomics signature designed for generalizability

Having laid out the foundation to construct a radiomic signature across centers, we sought to implement a global hyperparameter search framework (the “GHPS”) to determine the optimal combination of machine learning and imaging features to establish a final radiomics biomarker to predict PFS-6.

**Figure 4:**
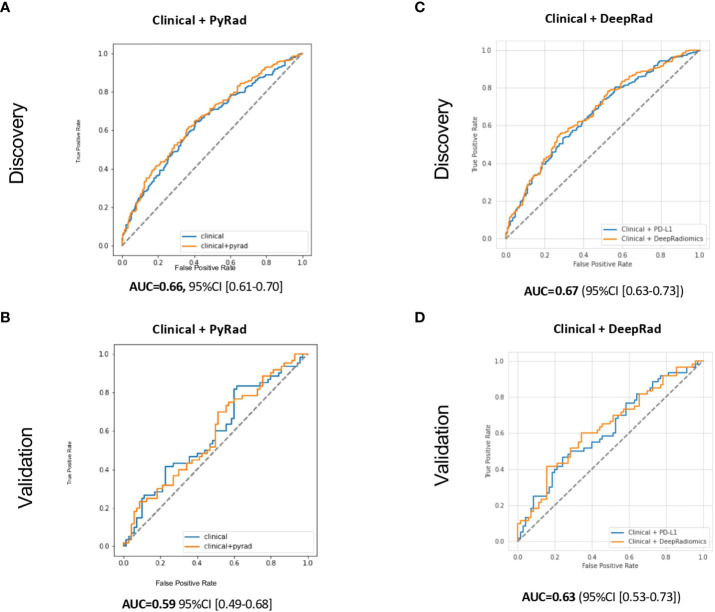
Receiver operating characteristic (ROC) curves for PFS-6 months prediction models with **(A)** Clinical (age, ECOG status, smoking status, and line of treatment) + PyRadiomics after harmonization, **(B)** Clinical + DeepRadiomics after harmonization in the discovery cohorts, **(C)** Clinical + PyRadiomics after harmonization, and **(D)** Clinical+ DeepRadiomics after harmonization in the validation cohorts. PyRad, PyRadiomics; DeepRad, DeepRadiomics.

First, using this construct, the combination of Clinical + PyRadiomics depicted an AUC of 0.66 (95% CI [0.61–0.70]) and 0.59 (95% CI [0.49–0.68]) in the discovery and validation cohorts, respectively, failing to meet the validation criteria for reproducibility (remaining within the discovery CI), *de facto* failing the generalizability objective (**Figures 4A, B**).

Second, the combination of Clinical + DeepRadiomics features reached an AUC of 0.67 (95% CI [0.63–0.73]) and 0.63 (95% CI [0.53–0.73]) in the discovery and validation cohort, respectively (**Figures 4C, D**). The validation cohort AUC of 0.63 also fell within its 95% CI estimate in the discovery cohort, meeting our generalizable objective. Interestingly, these results were comparable to Clinical + PD-L1 currently used in routine oncology practice. Moreover, both models also depicted a lower bound on the 95% CI of these models that was greater than 0.50 in the discovery cohort, confirming the predictive value of these models. Third, using both models, the addition of PD-L1 did not increase the performances (similar AUC) or the generalizability (**Supplementary Figures 5A–D**).

Finally, to provide valuable insights for future research to be leveraged as part of prior information for statistical study design, we conducted an exploratory assessment of non-inferiority using permutation analyses. Indeed, it is important to note that our study was not specifically designed or adequately powered for standard non-inferiority testing. Nonetheless, our findings indicate that the AUC of the Clinical + DeepRadiomics model was not statistically lower than the AUC of the Clinical + PD-L1 model (mean difference across permutations: 0.00035; *p*-value: 0.617*).*

## Discussion

Radiomics represent a promising non-invasive biomarker for patients amenable to ICI; however, generalizability especially in various centers represent the major limitation (10, 19). In this large study of advanced NSCLC treated with ICI across four institutions, we demonstrated that a risk prediction model that combined Clinical + DeepRadiomics was generalizable and was non-inferior to the Clinical + PD-L1 model currently used by oncologists to predict PFS-6 months.

Importantly, our results showed that, after generalizability, DeepRadiomics methods had a better performance than the PyRadiomics pipeline. This could be explained by our proposed combination of traditional harmonization techniques, which, together with a generalization-optimizing AI framework, overcomes these limitations of previous models that did not generalize and enables clinical utility. Our AI framework involves two automated steps: the discovery of relevant imaging features using DeepRadiomics that delivers informative, reproducible, and stable compressed representation of an imaging data space, and a global hyperparameter search that iteratively loops over all combination of the three methods used in our modeling process: (a) feature selection that chooses the best algorithm that identifies features to include in the model, maintaining the most “informative” features, and removing noisy “non-informative,” irrelevant and redundant features; (b) model selection that determines which machine learning estimator to use; and (c) hyperparameter tuning, which defines the optimum hyperparameter values to use for each estimator. While computationally intensive, this global search allows for the data-driven exploration of the somewhat unpredictable interplay between models and features (23). Consequently, we avoided radiomics features that were independently selected from other factors and/or not solely derived from the training portion of the data (in the machine learning training–validation–test sense), typically subject to an often-overlooked look-ahead bias and loss of future generalizability, addressing the problem of inappropriately applying cross-validation methods to feature selection (37).

However, the addition to our signature combining DeepRadiomics + Clinical was not improved by the implementation of a third parameter such as PD-L1. This could be explained by the limited discovery dataset available in this trial, which would otherwise be necessary to increase model parameters with sufficient examples for machine learning. Altogether, we obtained strong AUC in the discovery cohort independent of PD-L1 expression; however, there was no evidence of generalizability in the validation cohorts. This observation confirms that in the absence of homogeneous PCoA, radiomics validation is limited.

Furthermore, we acknowledge limitations in this study. First, although the CT scans were obtained from four institutions, only two physicians (a radiation oncologist and a radiologist) performed the image segmentations, reducing inter-observer variabilities. Second, the use of PyRadiomics is limited by the radiomic features being extracted from segmented ROI that required at least some degree of direct planimetry (and therefore additional physician time), subject to inter-annotator variability (38). This limitation was not present for the DeepRadiomics method we proposed, which does not require a segmentation input. Third, the validation cohort baseline characteristics had more patients treated with first line, which could decrease the performance of our model. Indeed, prior chemotherapy could impact the image features. Also, combination treatment such as chemotherapy with immunotherapy could modify the reproducibility of our model. Nevertheless, the DeepRadiomics method and the AI generalizability framework were able to mitigate this challenge. Lastly, with a primary objective to assess model generalizability, we eventually lacked the power to undertake non-inferiority testing. Our current results would indeed motivate further investigation in that direction, on another larger cohort of patients.

In conclusion, this radiomics generalizability study was able to demonstrate that a DeepRadiomics signature with harmonization developed in a discovery cohort from various centers could overcome the negative impact of variable CT acquisition parameters and then could be validated in an independent cohort. This DeepRadiomics harmonization signature warrants further improvement and validation in external cohorts of patients with NSCLC treated with ICI and opens a new non-invasive biomarker strategy.

## Data availability statement

The original contributions presented in the study are included in the article/**Supplementary Material**. Further inquiries can be directed to the corresponding author.

## Ethics statement

The studies involving human participants were reviewed and approved by MP-02-2019-8091. The patients/participants provided their written informed consent to participate in this study.

## Author contributions

MT annotated CT-scans, collected clinical data, and wrote and reviewed the manuscript. Imagia team (KP, CL-K, FD, LJ, FR, KK, DH, and FIC) completed statistical analysis, normalization, and radiomics features extraction. SK and JP collected clinical data. FrC annotated CT-scans. VM, DV JM, FIC, AG, WB, AE, MO, NB, TM, DH, PJ, and KP reviewed the manuscript. BR coordinated the project and reviewed the manuscript.

## Supplementary material

Supplementary Figure 1 Flow chart diagram of exclusion and final studied cohorts.

Supplementary Figure 2 Receiver operating characteristic (ROC) curves for the prediction models without radiomics and only with **(A)** clinical (age, ECOG status, smoking status, and line of treatment) + immunohistochemistry PD-L1 tumor expression, **(B)** representing clinical alone and **(C)** immunohistochemistry PD-L1 tumor expression in the discovery and **(D-F)** validation cohorts.

Supplementary Figure 3
**(A)** Receiver operating characteristic (ROC) curves for the prediction models with, clinical (age, ECOG status, smoking status, and line of treatment) + PyRadiomics before harmonization or clinical + DeepRadiomics before harmonization in the discovery cohorts **(B)** ROC curves of clinical + PyRadiomics before harmonization and clinical + DeepRadiomics before harmonization in the validation cohorts. **(C,D)** similar ROC curves but with the addiction of PD-L1 for the discovery and validation cohorts respectively.

Supplementary Figure 4
**(A)** Principal component analysis (PCoA) of DeepRadiomics features after normalization depicted by the VGG16 backbone network input of 24 pixels **(B)** PCoA of DeepRadiomics features stratified by outcome PFS-6 months.

Supplementary Figure 5 Receiver operating characteristic (ROC) curves for PFS-6 months prediction models with **(A)** Clinical (age, ECOG status, smoking status, and line of treatment) + PyRadiomics + PD-L1 after harmonization in the discovery cohort **(B)** Clinical + PyRadiomics + PD-L1 after harmonization in the validation cohort and **(C)** clinical + DeepRadiomics + PD-L1 after harmonization in the discovery cohort **(D)** clinical + DeepRadiomics + PD-L1 after harmonization in the validation cohort. PyRad, PyRadiomics; DeepRad, DeepRadiomics.

Supplementary Table 1 – Generalizability of the performance of the prediction models using Nagelkerke’s R, Bier’s score and area under the curve (AUC) as measures of AI-model calibration.

Supplementary Table 2 – Determination of the Sensitivity and Specificity of the Model in discovery and validation cohorts using the Yonden’s index.

Supplementary Table 3 - Baseline characteristics of computed tomography scan characteristics in the discovery and validation cohorts.
