# Supplementary figures and images for: Generalization optimizing machine learning to improve CT scan radiomics and assess immune checkpoint inhibitors’ response in non-small cell lung cancer: a multicenter cohort study
Source: Front Oncol. 2023 Jul 20;13:1196414. doi: 10.3389/fonc.2023.1196414 (PMC10400292; doi:10.3389/fonc.2023.1196414)

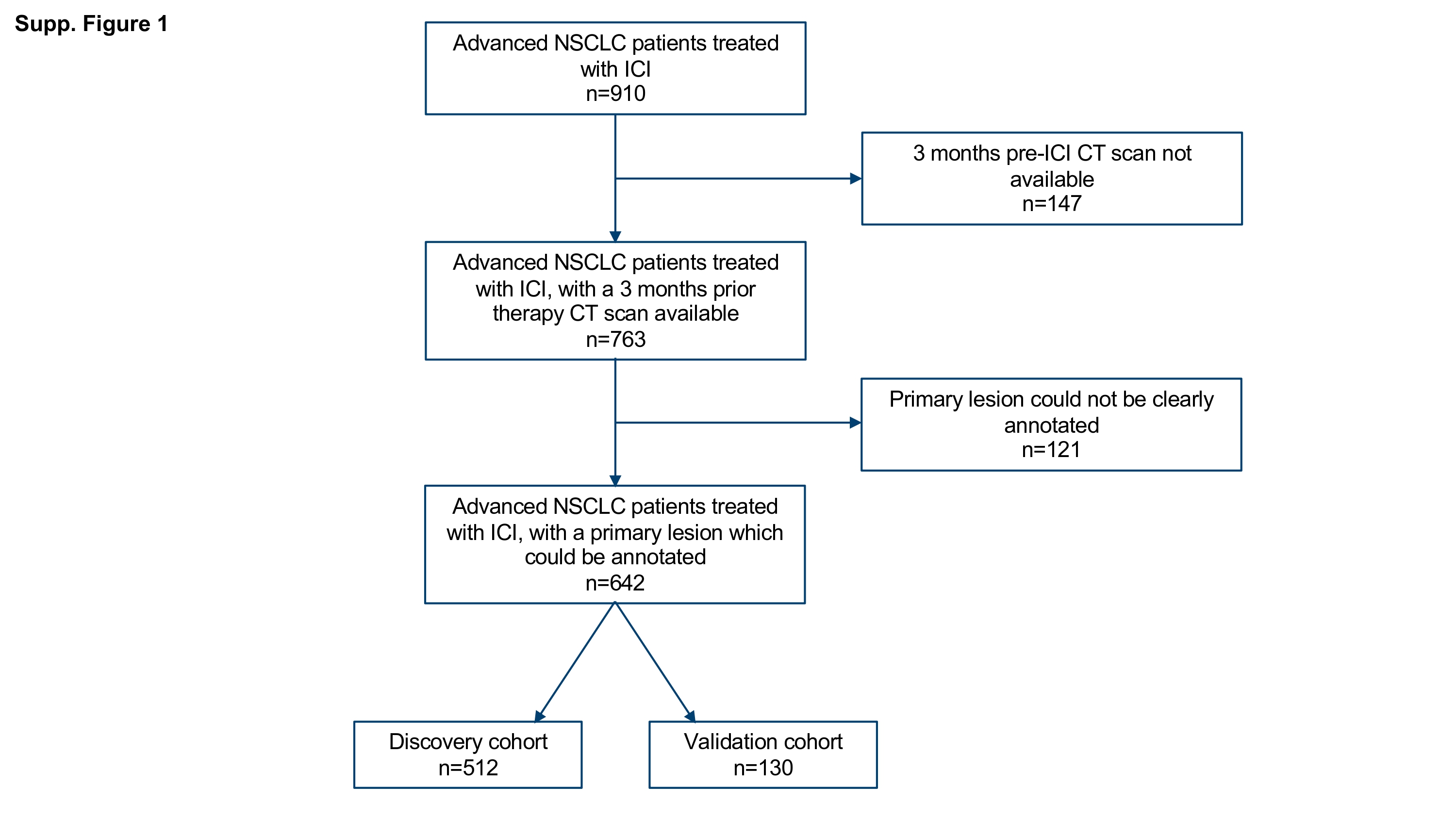

Supplement: Supplementary Figure 1 — Flow chart diagram of exclusion and final studied cohorts. [file Image_1.jpeg]

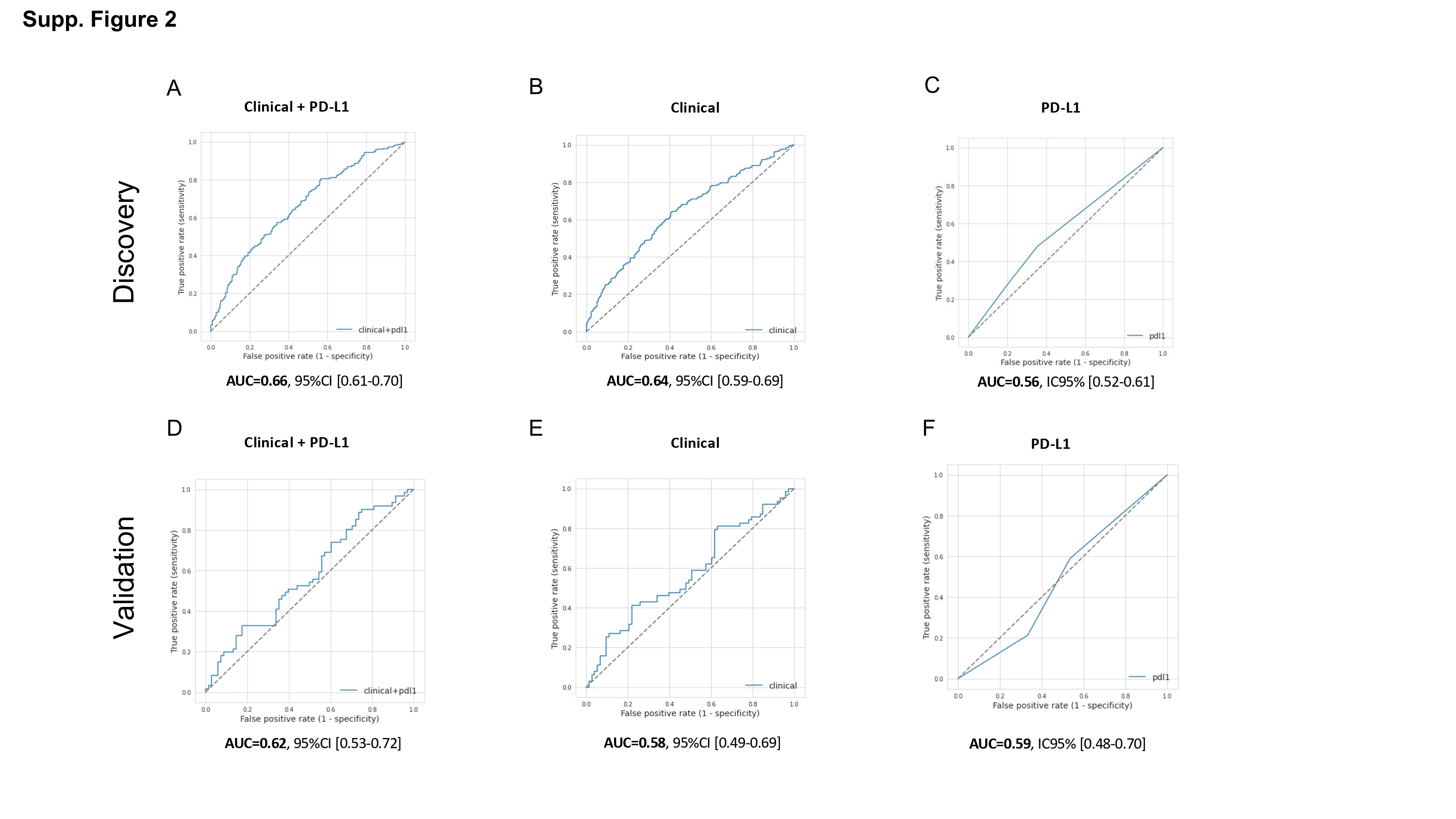

Supplement: Supplementary Figure 2 — Receiver operating characteristic (ROC) curves for the prediction models without radiomics and only with (A) clinical (age, ECOG status, smoking status, and line of treatment) + immunohistochemistry PD-L1 tumor expression, (B) representing clinical alone and (C) immunohistochemistry PD-L1 tumor expression in the discovery and (D-F) validation cohorts. [file Image_2.jpeg]

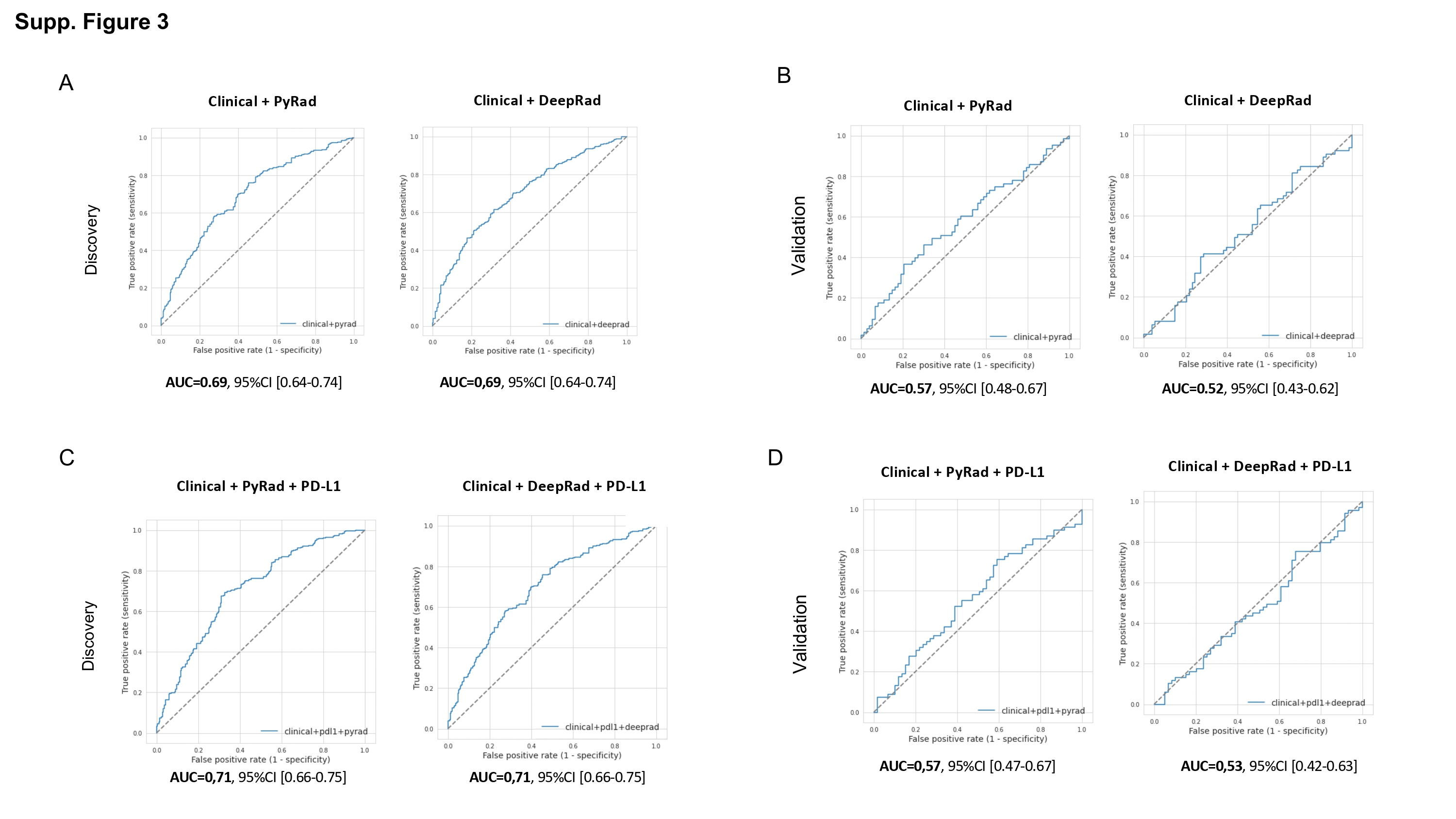

Supplement: Supplementary Figure 3 — (A) Receiver operating characteristic (ROC) curves for the prediction models with, clinical (age, ECOG status, smoking status, and line of treatment) + PyRadiomics before harmonization or clinical + DeepRadiomics before harmonization in the discovery cohorts (B) ROC curves of clinical + PyRadiomics before harmonization and clinical + DeepRadiomics before harmonization in the validation cohorts. (C,D) similar ROC curves but with the addiction of PD-L1 for the discovery and validation cohorts respectively. [file Image_3.jpeg]

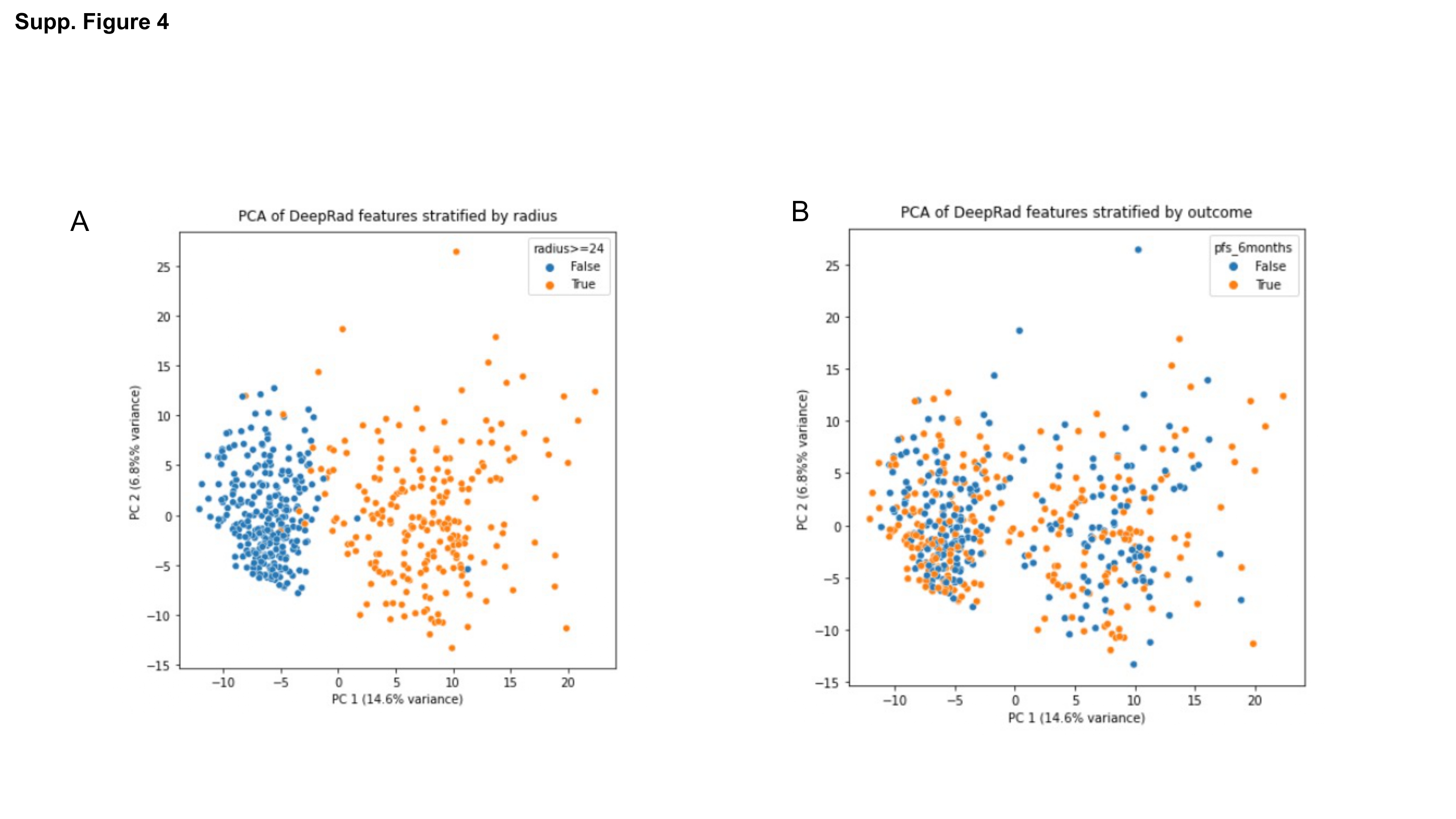

Supplement: Supplementary Figure 4 — (A) Principal component analysis (PCoA) of DeepRadiomics features after normalization depicted by the VGG16 backbone network input of 24 pixels (B) PCoA of DeepRadiomics features stratified by outcome PFS-6 months. [file Image_4.jpeg]

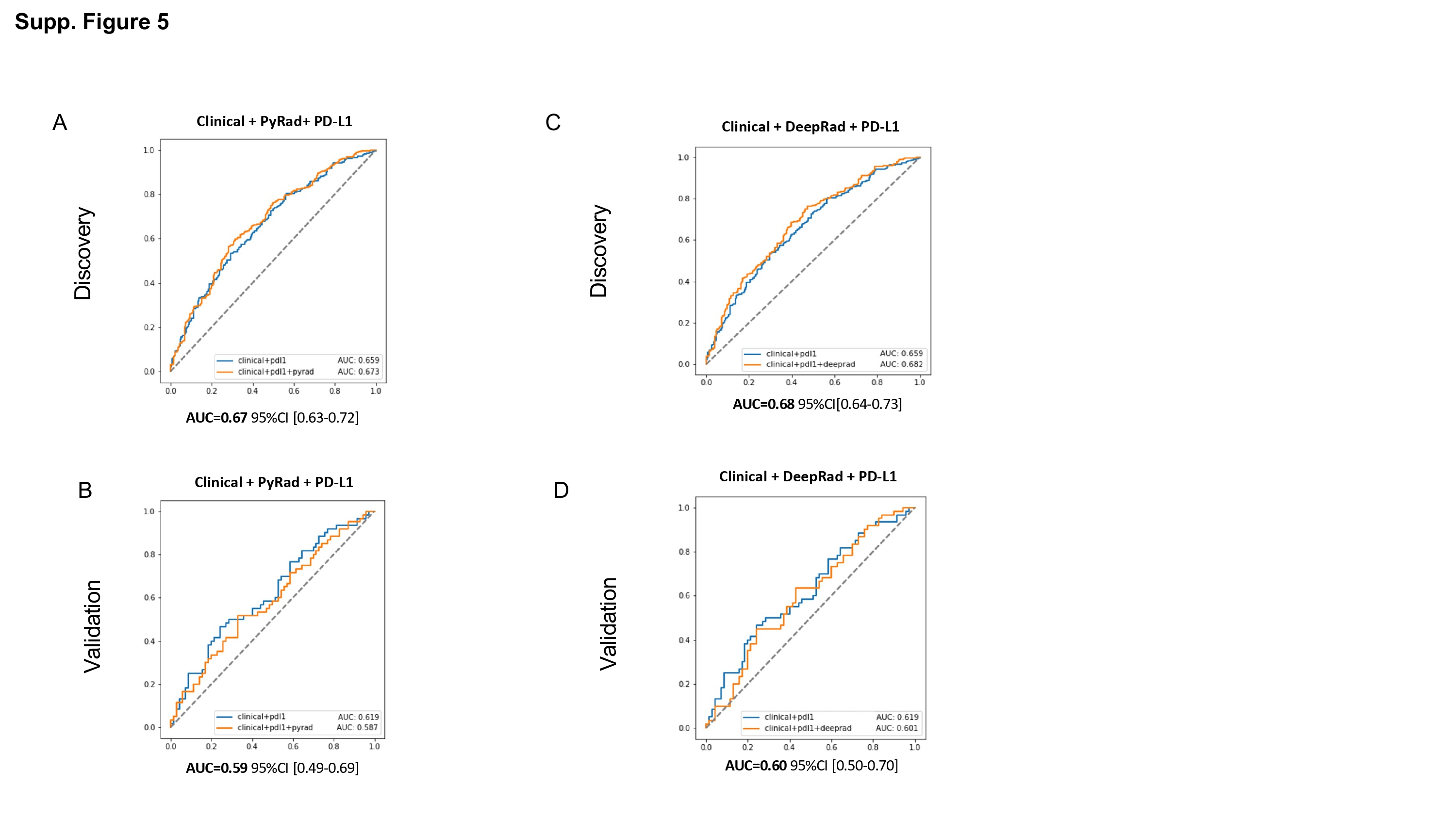

Supplement: Supplementary Figure 5 — Receiver operating characteristic (ROC) curves for PFS-6 months prediction models with (A) Clinical (age, ECOG status, smoking status, and line of treatment) + PyRadiomics + PD-L1 after harmonization in the discovery cohort (B) Clinical + PyRadiomics + PD-L1 after harmonization in the validation cohort and (C) clinical + DeepRadiomics + PD-L1 after harmonization in the discovery cohort (D) clinical + DeepRadiomics + PD-L1 after harmonization in the validation cohort. PyRad, PyRadiomics; DeepRad, DeepRadiomics. [file Image_5.jpeg]
