# Supplementary material for: Generalization optimizing machine learning to improve CT scan radiomics and assess immune checkpoint inhibitors’ response in non-small cell lung cancer: a multicenter cohort study
Source: Front Oncol. 2023 Jul 20;13:1196414. doi: 10.3389/fonc.2023.1196414 (PMC10400292; doi:10.3389/fonc.2023.1196414)
Supplement: Supplementary Table 1 — – Generalizability of the performance of the prediction models using Nagelkerke’s R, Bier’s score and area under the curve (AUC) as measures of AI-model calibration. [file Table_1.pdf]

Supp. Table 1

		Nagelkerke's R^2	Brier's score	AUC
**Discovery cohort**	**Clinical + PD-L1**	0.10 [0.05-0.16]	0.23 [0.22-0.24]	0.66 (0.61-0.70)
	**Clinical + PyRadiomics**	0.10 [0.04-0.15]	0.23 [0.22-0.24]	0.66 (0.61-0.70)
	**Clinical + DeepRadiomics**	0.12 [0.06-0.19]	0.23 [0.21-0.24]	0.67 (0.63-0.72)
**Validation cohort**	**Clinical + PD-L1**	0.03 [-0.08-0.15]	0.24 [0.22-0.26]	0.63 (0.53-0.72)
	**Clinical + PyRadiomics**	0.03 [-0.06-0.12]	0.24 [0.23-0.26]	0.59 (0.49-0.68)
	**Clinical + DeepRadiomics**	0.08 [-0.04-0.18]	0.23 [0.21-0.25]	0.63 (0.54-0.73)
