# Supplementary material for: Generalization optimizing machine learning to improve CT scan radiomics and assess immune checkpoint inhibitors’ response in non-small cell lung cancer: a multicenter cohort study
Source: Front Oncol. 2023 Jul 20;13:1196414. doi: 10.3389/fonc.2023.1196414 (PMC10400292; doi:10.3389/fonc.2023.1196414)
Supplement: Supplementary Table 2 — – Determination of the Sensitivity and Specificity of the Model in discovery and validation cohorts using the Yonden’s index. [file Table_2.pdf]

Supp. Table 2

	Model	Youden's J	Sensitivity	Specificity
**Discovery**	Clinical + PD-L1	0.26 [0.19-0.33]	0.65 [0.45-0.83]	0.61 [0.42-0.78]
	Clinical + PyRadiomics	0.26 [0.19-0.34]	0.61 [0.39-0.74]	0.66 [0.51-0.86]
	Clinical + DeepRadiomics	0.29 [0.21-0.36]	0.59 [0.50-0.83]	0.71 [0.45-0.78]
**Validation**	Clinical + PD-L1	0.25 [0.10-0.39]	0.55 [0.33-0.90]	0.73 [0.31-0.89]
	Clinical + PyRadiomics	0.21 [0.07-0.36]	0.72 [0.25-0.90]	0.51 [0.27-0.96]
	Clinical + DeepRadiomics	0.29 [0.13-0.42]	0.57 [0.37-0.80]	0.72 [0.39-0.90]
