# Supplementary material for: Generalization optimizing machine learning to improve CT scan radiomics and assess immune checkpoint inhibitors’ response in non-small cell lung cancer: a multicenter cohort study
Source: Front Oncol. 2023 Jul 20;13:1196414. doi: 10.3389/fonc.2023.1196414 (PMC10400292; doi:10.3389/fonc.2023.1196414)
Supplement: Supplementary Table 3 — - Baseline characteristics of computed tomography scan characteristics in the discovery and validation cohorts. [file Table_3.pdf]

Supp. Table 3

	Discovery cohort n=512	Validation cohort n=130	*p-value*
**Manufacturer** - n (%)			
GE Medical Systems	92 (18.0)	28 (21.7)	*<0.001*
Philips	64 (12.5)	2 (1.6)	
Siemens	336 (65.6)	10 (7.8)	
Toshiba	20 (3.9)	89 (69.0)	
**Convolution kernel** - n (%)			
Sharp	283 (55.3)	126 (96.9)	*<0.001*
Soft	229 (44.7)	4 (3.1)	
**Slice thickness** - n (%)			
[0-1 mm]	15 (2.9)	0 (0)	*<0.001*
[1-2 mm]	418 (81.6)	59 (45.7)	
[2-5 mm]	79 (15.4)	70 (54.3)	
**Kilo Volt Peak (kVp)** - n (%)			
80	1 (0.2)	0 (0)	*0.004*
100	166 (32.4)	62 (48.1)	
120	345 (67.4)	67 (51.9)	
**Exposure** - median mA [IQR]	106.8 [57.1,146.8]	116.0 [72.0,143.0]	*0.522*
**X-ray tube current** - median mA [IQR]	232.0 [161.4,316.0]	263.0 [199.0,304.0]	*0.054*
